# Pareidolia in Schizophrenia and Bipolar Disorder

**DOI:** 10.3389/fpsyt.2021.746734

**Published:** 2021-12-10

**Authors:** Eid G. Abo Hamza, Szabolcs Kéri, Katalin Csigó, Dalia Bedewy, Ahmed A. Moustafa

**Affiliations:** ^1^Psychology Department, College of Humanities and Sciences, Ajman University, Ajman, United Arab Emirates; ^2^College of Education, Tanta University, Tanta, Egypt; ^3^National Institute of Psychiatry and Addictions, Budapest, Hungary; ^4^Department of Cognitive Science, Budapest University of Technology and Economics, Budapest, Hungary; ^5^Department of Physiology, University of Szeged, Szeged, Hungary; ^6^Department of Human Anatomy and Physiology, the Faculty of Health Sciences, University of Johannesburg, Johannesburg, South Africa; ^7^School of Psychology & Marcs Institute for Brain and Behaviour, Western Sydney University, Sydney, NSW, Australia

**Keywords:** psychosis, visual illusion, pareidolia, bipolar disorder, schizophrenia

## Abstract

While there are many studies on pareidolia in healthy individuals and patients with schizophrenia, to our knowledge, there are no prior studies on pareidolia in patients with bipolar disorder. Accordingly, in this study, we, for the first time, measured pareidolia in patients with bipolar disorder (*N* = 50), and compared that to patients with schizophrenia (*N* = 50) and healthy controls (*N* = 50). We have used (a) the scene test, which consists of 10 blurred images of natural scenes that was previously found to produce illusory face responses and (b) the noise test which had 32 black and white images consisting of visual noise and 8 images depicting human faces; participants indicated whether a face was present on these images and to point to the location where they saw the face. Illusory responses were defined as answers when observers falsely identified objects that were not on the images in the scene task (maximum illusory score: 10), and the number of noise images in which they reported the presence of a face (maximum illusory score: 32). Further, we also calculated the total pareidolia score for each task (the sum number of images with illusory responses in the scene and noise tests). The responses were scored by two independent raters with an excellent congruence (kappa > 0.9). Our results show that schizophrenia patients scored higher on pareidolia measures than both healthy controls and patients with bipolar disorder. Our findings are agreement with prior findings on more impaired cognitive processes in schizophrenia than in bipolar patients.

## Introduction

Schizophrenia (SCZ) is a psychiatric disorder characterized by positive and negative symptoms (1). Positive symptoms involve delusions and hallucinations, while negative symptoms include avolition and diminished emotional expression. Bipolar disorder (BPD) is a mood disorder characterized by alternating states of depression and mania or hypomania (1). As we discuss below, both disorders show both similar and dissimilar kinds of perceptual, cognitive, and neural patterns.

Studies have shown that patients with bipolar disorder and schizophrenia show both similar and dissimilar neural abnormalities [for discussion on this point, see (2–4)]. For example, one study reported that genes underlying dopamine function in the prefrontal cortex are similarly implicated in both schizophrenia and bipolar disorder (5). Parker et al. (6) found that the neural similarities in both schizophrenia and bipolar disorder depends on severity of psychosis in bipolar disorder. Unlike bipolar patients without psychosis, bipolar disorder patients with psychosis tends to show similar EEG responses (particularly P50) to schizophrenia. Given that several studies show that patients with bipolar disorder show milder forms of cognitive dysfunction in comparison to patients with schizophrenia (7), it is expected that schizophrenia patients will show more widespread and severe neural damage compared to patients with bipolar disorder. Confirming this, it has been reported that while patients with bipolar disorder and schizophrenia show both prefrontal and striatal damage (8–10), these abnormalities are pronounced in patients with schizophrenia than in patients with bipolar disorder (11). In sum, neural studies have shown that patients with bipolar disorder show milder neural abnormalties than patients with schizophrenia and that severity of psychosis in bipolar disorder is associated with more accentuated neural damage.

### Perception, Visual Illusion, and Pareidolia

Our perception of the world is not solely determined by the input to our senses, but is strongly influenced by our prior experience with the world. A visual percept is inferred from often fragmentary and incomplete visual signals from the eyes through a process of unconscious inference (12). For example, a face emerging from the shadows, which is barely visible, can be recognized despite only a small patch of light being received by the retina. The rest of the face is inferred based on prior knowledge, expectations, and our beliefs about faces. Illusory face detection is common in the human population (e.g., detection rates as high as 41%) (13); however, research suggests substantial individual variation (14).

Several studies suggest that illusion can result from deficits in top-down processing, that is, existing cognitive and perceptual biases (15–19) or an imbalance between top-down internal factors (e.g., perceptual expectations, prior knowledge, and mental imagery) and bottom-up external sensory input (15, 20). Moreover, sensory isolation can induce the same effect as a perceptual bias toward prior expectations that leads to visual illusion (21, 22).

Auditory illusion, often known as auditory misperception or hallucinations, is defined as perceiving sounds that do not exist in the presented stimuli, is common in schizophrenia. Auditory illusion has been reported using several experimental paradigms (23–25). Thus, a better understanding of the underlying cause may help treat such symptoms and prevent such outcomes. Typically, illusion in schizophrenia patients include people, faces, animals, objects with frightening content (26–28). Like schizophrenia, patients with bipolar disorder also show visual illusion (29, 30). However, compared to schizophrenia, there are fewer studies on visual hallucinations and illusions in bipolar disorder (29, 31). Prior studies have found that patients with bipolar disorder are similar to healthy controls in terms of experiencing illusion. For example, Keane et al. (32) found that patients with bipolar disorders and healthy controls show normal depth inversion illusions, while patients with schizophrenia show reduced depth inversion illusions. It is important to note that unlike other forms of illusions, experiencing depth inversion illusion is considered normal and common, and not experiencing this form of illusion is associated with neural abnormalities in the parietal-frontal network (33). However, some studies found that both patients with bipolar disorder and schizophrenia show similar performance in the Mueller-Lyer illusion task (30, 34).

Pareidolia is the perception of faces in ambiguous visual stimuli, such as clouds, rock formations, or flocks of birds, and is thus a type of visual illusion (35). Pareidolia occurs when an indistinct and often randomly formed stimulus is interpreted as being definite and meaningful. This is something that many individuals have experienced, whether exercising their imagination as a cloud-gazing child, or seeing images in a textured ceiling during the last few waking moments of the day.

Several studies show that healthy “normal” people report pareidolic experiences. Uchiyama et al. (36) found that pareidolia is related to impaired visual and perceptual processes. There have been several studies investigated personality traits and individual differences in relation to pareidolia (37). It was reported that pareidolia is high in religious individuals (38) and individuals high in schizotypy (39). Other studies found that mood states and feeling lonely may increase the occurrence of pareidolia (40). Pareidolic experiences are commonly reported during the use of hallucinogens such as Lysergic Acid Diethylamide (LSD) in healthy individuals [for discusssion see, (41)]. One recent ERP study has investigated the occurrence of pareidolia in healthy individuals, showing that some EEG components can differentiate faces from face pareidolia (42). In this study, N170 was larger for faces than face pareidolia, but VPP was larger for face pareidolia than for faces. Using fMRI, Wardle et al. (43) found that face pareidolia is associated with the activation of fusiform area.

In addition, there have been few studies investigating pareidolia in patient populations including autism (44), patients with migraine (45), schizophrenia (46), Lewy Bodies Dementia (36), and Parkinson's disease (47). To our knowledge, there are only two study investigating pareidolia in schizophrenia (46, 48), and no study has investigated pareidolia in bipolar disorder.

### The Current Study

The current study has, for the first time, investigated and compared pareidolia measures in both schizophrenia and bipolar disorder patients, using tasks that were not previously used with these patient populations. In addition, we have also used several other clinical measures including the Structured Interview for Assessing Perceptual Anomalies (SIAPA) to measure both auditory and visual misperceptions.

## Methods

Below, we describe the characteristics of our participants, pareidolia tests, and statistical analyses. The study was approved by the Hungarian Scientific and Research Committee of the Medical Research Council ethics board (Budapest, Hungary).

### Participants

We enrolled 50 patients with schizophrenia, 50 patients with type I bipolar disorder with a history of psychotic symptoms, and 50 control volunteers without any history of psychiatric disorders. Participants were matched for age, sex, education, Intelligence Quotient (IQ), and general psychosocial functions (Table 1). The study was coordinated in the Nyíro Gyula National Institute of Psychiatry and Addictions and was approved by the Hungarian Scientific and Research Committee of the Medical Research Council ethics board (Budapest, Hungary). All participants gave written informed consents. The inclusion criteria are as follows: Diagnostic and Statistical Manual of Mental Disorders (DSM-5) diagnosis of bipolar disorder or schizophrenia, ability and willingness to participate, age between 18 and 65 years, and lack of acute psychosis. All of our participants were outpatients. All patients lived in the community and were in clinical remission according to the Andreasen-criteria for schizophrenia (49) and to the Systematic Treatment Enhancement Program for Bipolar Disorder criteria for bipolar disorder (50).

**Table 1:** Clinical and demographical characteristics of the participants.

	**Schizophrenia (SCZ) (*n* = 50)**	**Bipolar disorder (BPD) (*n* = 50)**	**Control participants (*n* = 50)**
Age (years)	39.5 (8.4)	40.5 (7.9)	38.2 (8.5)
Sex (male/female)	32/18	32/18	32/18
Education (years)	11.8 (5.7)	11.7 (6.1)	12.0 (6.3)
IQ	98.4 (9.5)	100.5 (9.0)	101.7 (10.4)
Visual acuity (LogMAR)	0.17 (0.09)	0.19 (0.08)	0.17 (0.04)
Duration of Illness (years)	14.7 (8.3)	14.9 (9.1)	–
WHODAS 2.0	21.3 (7.9)	20.4 (6.8)	–
PANSS—P	15.7 (5.2)	16.8 (5.8)	–
PANSS—N*	18.1 (4.8)	15.4 (5.1)	–
PANSS—G	40.2 (10.1)	39.2 (12.1)	–
HAM-D	10.3 (5.8)	10.9 (7.5)	–
YMRS	4.5 (2.1)	4.0 (2.9)	–
Chlorpromazine (CPZE)-equivalent antipsychotic dose (mg/day)	326.3 (179.4)	328.7 (215.5)	–

Data are mean (standard deviation) except the sex ratio. PANSS, Positive and Negative Syndrome Scale; HAM-D, Hamilton Rating Scale for Depression; YMRS, Young Mania Rating Scale; WHODAS 2.0, WHO Disability Assessment Schedule 2.0; ^*^p < 0.05 (two-tailed t-test).

The exclusion criteria were as follows: neurological disorders and other general medical conditions affecting the central nervous system, evidence of head injury, electroconvulsive therapy, and psychoactive substance misuse confirmed by clinical history or by a urine test. In total, we have excluded only five patients with comorbid substance and alcohol misuse. Forty-seven patients with schizophrenia and 40 patients with bipolar disorder received either second-generation antipsychotic medications (amisulpride, olanzapine, quetiapine, and risperidone) or third-generation medication (e.g., aripiprazole) at the time of testing. In total, 32 schizophrenia patients, 34 bipolar disorder patients, and 30 controls regularly smoked tobacco. The chlorpromazine-equivalent doses, calculated by a standard method (51), are shown in Table 1. Twelve patients with schizophrenia and 45 patients with bipolar disorder also received mood stabilizers (lithium, valproate, or lamotrigine).

### Pareidolia Tests and Clinical Measures

The pareidolia tests were based on the exact adoption of a previously published protocol (36, 52–54). The *scene test* consisted of 10 blurred images of natural scenes that frequently produced illusory face responses in a previous study (36, 53). The task goal was to point to and describe the objects on each image in as much detail as possible. The *noise pareidolia test* included 32 black and white images consisting of visual noise (spatial frequency: 1/f^3^) and 8 images depicting human faces. Participants were asked to respond whether a face was present on these images and to point to the location where they saw the face. The maximum exposure time was 60 s in the scene task and 30 s in the face task. Participants did not receive feedback on the appropriateness of their responses, and they were not informed that in the face task only noise was presented. Illusory responses were defined as answers when observers falsely identified objects that were not on the images in the scene task (maximum illusory score: 10), and the number of noise images in which they reported the presence of a face (maximum illusory score: 32). We also calculated the total pareidolia score for each task (the sum number of images with illusory responses in the scene and noise tests). The responses were scored by two independent raters with an excellent congruence (kappa > 0.9).

We used the following instruments for clinical evaluation: Structured Clinical Interview for DSM-5 Disorders—Clinician Version (SCID-5-CV) (55), Positive and Negative Syndrome Scale (PANSS) (56), Hamilton Rating Scale for Depression (HAM-D) (57), Young Mania Rating Scale (YMRS) (58), and the World Health Organization Disability Assessment Schedule (WHODAS 2.0) of the DSM-5 (1).

General intellectual and cognitive functions were measured with the Wechsler Adult Intelligence Scale-IV (WAIS-IV) (59). The DSM-5 structured clinical interview and the rating scales were administered by trained and supervised clinical psychologists or psychiatrists. Below, we provide a brief description of WHODAS 2.0, HAM-D, and PANSS scales.

### WHODAS 2.0

This instrument enables assessment of health and disability in 6 domains (cognition, mobility, self-care, interacting with other people, life activities, participation in communities). The administration time is short (5 to 20 min), and WHODAS 2.0. is valid in clinical and general populations across cultures. The concept of WHODAS 2.0. is based on ICF (International Classification of Functioning, Disability and Health) principles.

### HAM-D

We used the structured version of the 17-item HAM-D to assess the severity of depressive symptoms (e.g., depressed mood, feelings of guilt, suicide, and insomnia). Each item was rated on a 3-point (items 4–6, 12–14, 17) or 5-point Likert-scale (0—absent or no difficulty). A score in the range of 0–7 is normal. Patients scoring 20 or higher regularly require clinical attention.

### PANSS

The patient is scored from 1 to 7 on 30 items classified according to positive, negative, and general symptoms. The positive symptom scale contains 7 items (minimum score = 7, maximum score = 49), such as delusions, conceptual disorganization, hallucinations, excitement, and grandiosity. The negative scale also consists of 7 items (e.g., blunted affect, emotional withdrawal, and poor rapport). Finally, the general psychopathology scale consists of 16 items (minimum score = 16, maximum score = 112) (e.g., somatic concerns, anxiety, guilt, and tension).

### YMRS

The scale includes 11 items. Four items are graded on a 0 to 8 scale (irritability, speech, thought content, and disruptive or aggressive behavior), whereas seven items are graded on a 0 to 4 scale (e.g., elevated mood, increased motor activity, and sexual interest). A score of 20 or higher indicate severe mania.

#### Interview for the Assessment of Anomalous Visual and Auditory Experiences

We used the Structured Interview for Assessing Perceptual Anomalies (SIAPA) (60, 61). The SIAPA focuses on three aspects of anomalous perceptual experiences: sensory intensity (hypersensitivity), inundation or flooding, and selective attention to external stimuli on a scale of 0 (absent)−4 (pervasive). The interview begins with open-ended and then structured questions regarding subjective experiences in each sensory modality. For example, to assess hypersensitivity in the auditory modality, the following questions are presented: “Have you ever had the feeling or sensation that sounds were particularly loud? Or louder than usual? Or that your sense of hearing was particularly keen or sensitive? Or that your ears were picking up the slightest detail of sounds?” To evaluate inundation and flooding, participants are asked: “Have you ever had the experience or felt like you were being flooded or inundated by sounds? Or that you couldn't block out sounds? Or that it seemed as if your ears were picking up everything going on around you?” Finally, questions for selective attention are as follows: “Have you ever had the experience or felt like you couldn't pay attention to one sound, or a conversation, because of interference from other sounds, like background noise? Do you find that your attention is captured by irrelevant sounds, like traffic noises, even though they are of no interest to you?” (60). Similarly, in the visual modality, participants are asked whether lights seemed much brighter, colors were unusually vivid, the environment was bothersome, and whether they were overwhelmed by multiple objects in the scene and could not attend to one of many simultaneous visual inputs. We observed a good congruence between two independent raters (kappa > 0.7), and the internal consistency was good (Cronbach alpha > 0.8). The SIAPA scores correlated with objective psychophysical measures of sensory perception (61).

### Statistical Analysis

The STATISTICA 13.1 (Tibco, Palo Alto) software package was used for data analysis. First, we tested data distribution and homogeneity of variance with Lilliefors and Levene's tests, respectively. Measures with normal distributions were entered into analyses of variance (ANOVAs) and two-tailed Student's *t*-tests. Dichotomous variables were analyzed with chi-square tests. The SIAPA and the pareidolia scores were not normally distributed (*p* < 0.01), and therefore we used Kruskal-Wallis analyses of variance (ANOVA) followed by multiple comparisons for mean ranks tests. Spearman's rank correlation coefficients were calculated between the pareidolia scores and the clinical measures. We used a receiver operating characteristic (ROC) analysis to test the sensitivity and specificity of the pareidolia test to differentiate schizophrenia from bipolar disorder or controls. The schizophrenia-control and schizophrenia- bipolar disorder differentiation was also investigated with discriminant function analyses. The level of statistical significance was set at alpha <0.05, corrected for multiple comparisons with the Bonferroni method.

## Results

### The Pareidolia Test

**Figure 1:**
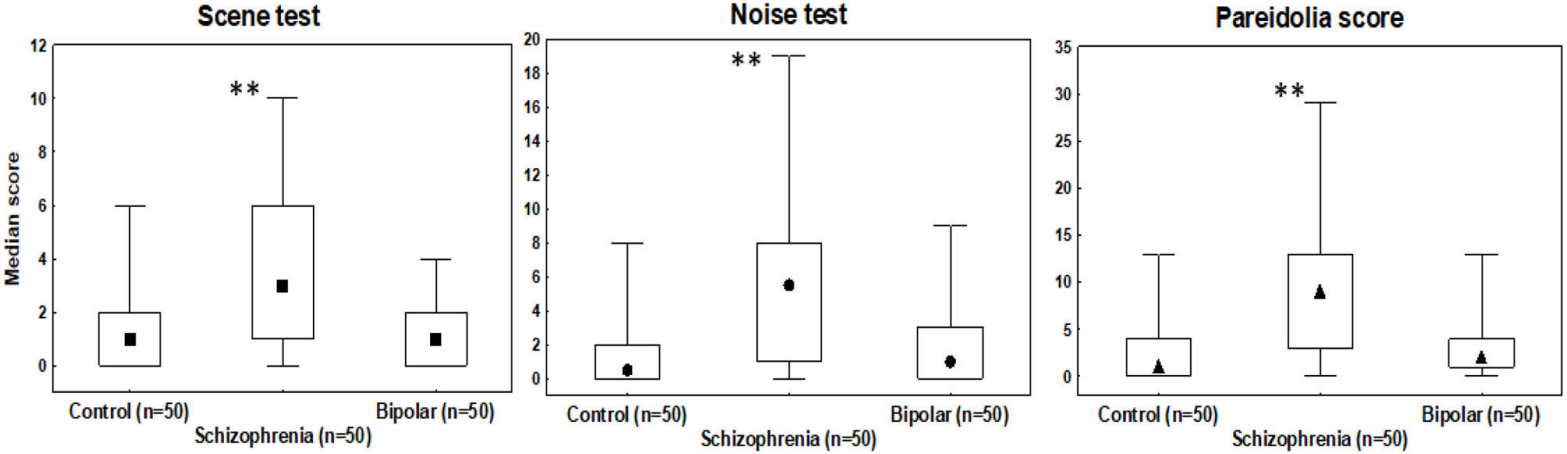
Median scores in the scene and noise test, and in the total pareidolia measures. Error bars indicate range; boxes indicate 25–75% percentiles. **p < 0.001, schizophrenia patients outscoring controls and patients with bipolar disorder.

Kruskal-Wallis ANOVAs that were conducted on the number of illusory responses indicated a significant difference among patients with schizophrenia, bipolar disorder, and controls in the scene test [H (2) = 31.59, *p* < 0.001], in the noise test [H (2) = 29.68, *p* < 0.001], and in the total pareidolia score [H (2) = 33.30, *p* < 0.001]. *Post-hoc* tests indicated that patients with schizophrenia scored higher than controls and patients with bipolar disorder on all measures of pareidolia (*p*'s < 0.01) (Figure 1). In contrast, we observed no significant between-group differences in the number of correctly identified faces in the noise task (*p* = 0.52; median: 8 in each group with a lower-upper quartile of 7–8).

**Figure 2:**
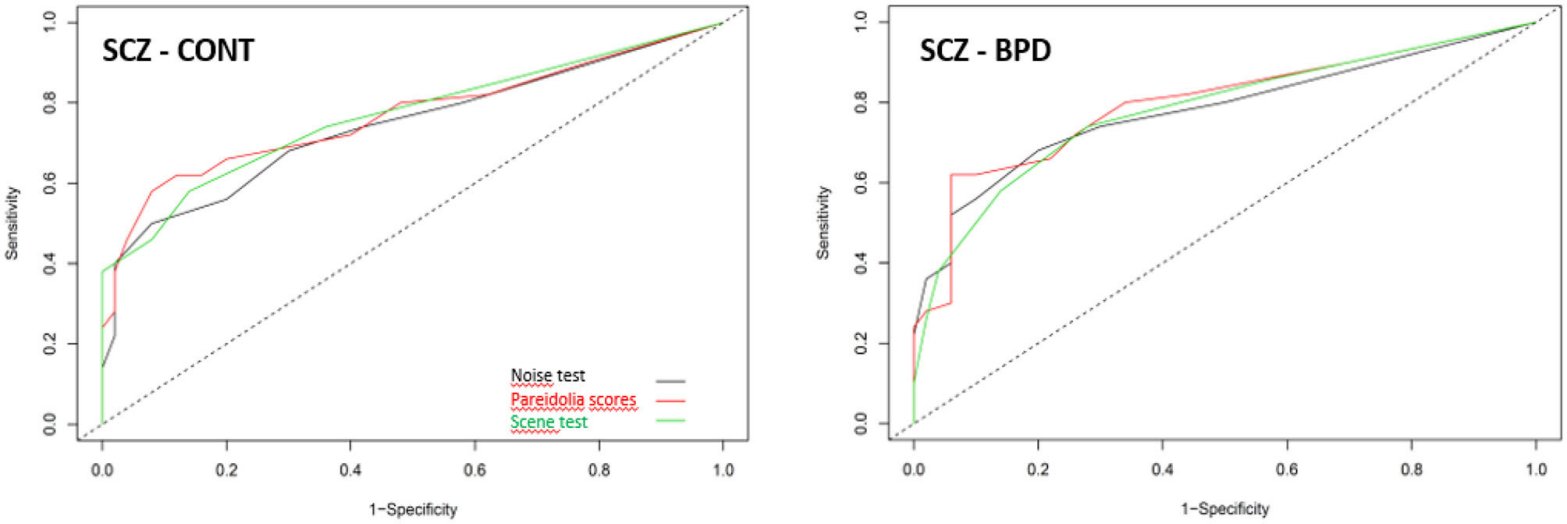
Receiver operating characteristic analysis for the pareidolia test to differentiate schizophrenia (SCZ) from controls (CONT) and schizophrenia from bipolar disorder (BPD).

Pareidolia measures differentiated schizophrenia from controls with a sensitivity of 74% (scene test) and a specificity of 94% (total pareidolia score). In the schizophrenia—bipolar disorder differentiation, the highest sensitivity was 62% (total pareidolia score) and the highest specificity was 92% (noise test). The results from the ROC analysis are summarized in Table 2 and Figure 2.

**Table 2:** Results from the ROC analysis.

	**SCZ—Control**				**SCZ—BPD**			
	**Sensitivity**	**Specificity**	**AUC**	**Cut-off**	**Sensitivity**	**Specificity**	**AUC**	**Cut-off**
Scene test	0.74	0.72	0.78	2	0.58	0.86	0.77	3
Noise test	0.68	0.80	0.78	3	0.50	0.92	0.74	6
Pareidolia score	0.62	0.94	0.80	7	0.62	0.88	0.77	7 8

*Receiver operating characteristic (ROC) analysis using an optimal cut-off score set (Youden-method). SCZ, schizophrenia; BPD, bipolar disorder; AUC, area under the curve*.

Discriminant function analysis also indicated a significant difference between schizophrenia and controls in all tests (see Supplementary Table 1). Altogether, 76% of the cases were correctly classified by using the total pareidolia scores. In the schizophrenia- bipolar disorder discrimination, the most successful classification was also observed in the case of the total pareidolia scores (73% of correctly classified cases). In contrast, we observed no significant effects in the schizophrenia- bipolar disorder discrimination with a maximum of 57% of correctly classified cases (Supplementary Table 1).

### Anomalous Perceptual Experiences (SIAPA) and Pareidolia

**Figure 3:**
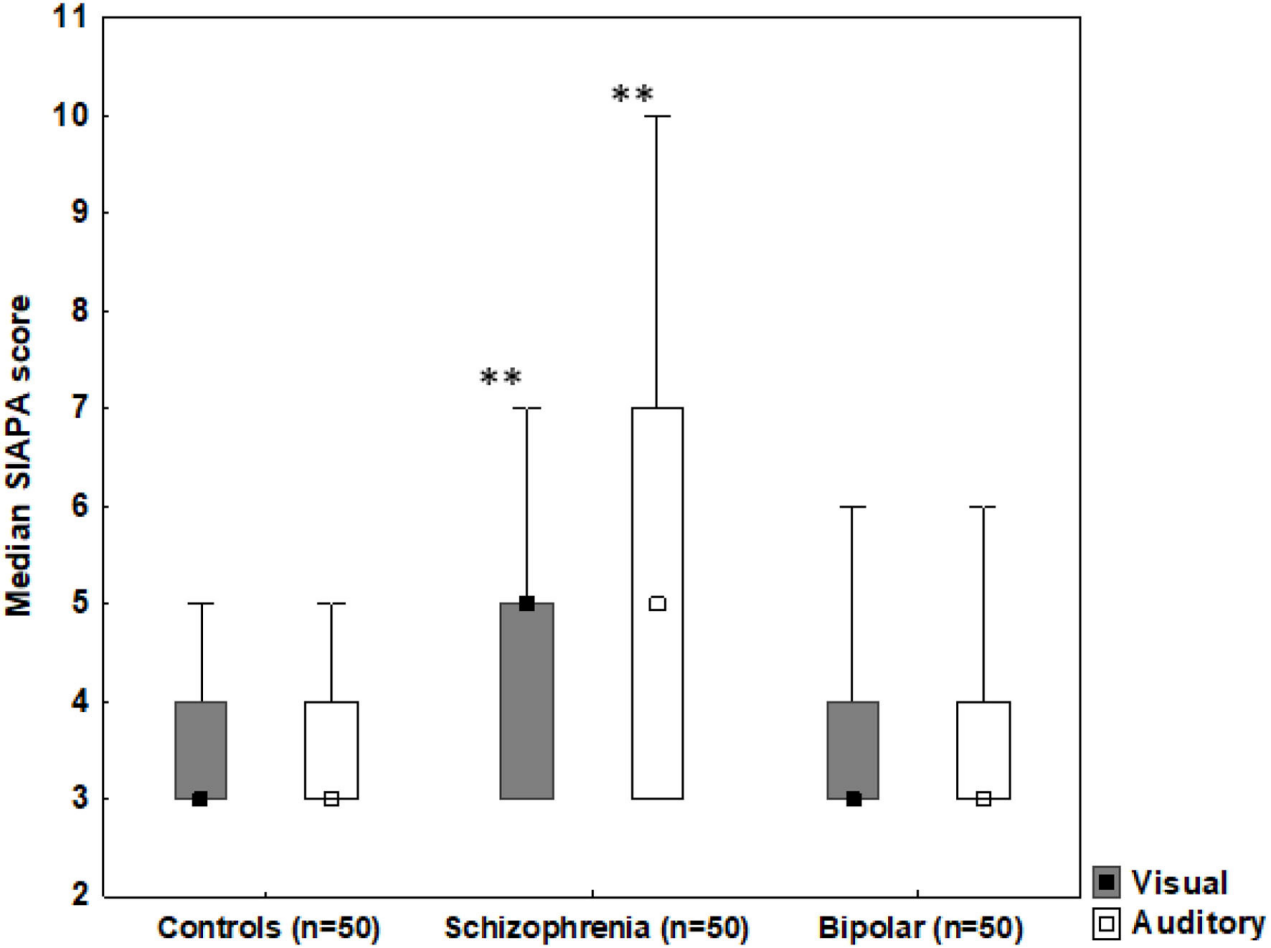
Median SIAPA (Structured Interview for Assessing Perceptual Anomalies) scores from the visual and auditory modalities. Error bars indicate range, boxes indicate 25–75% percentiles. **p < 0.001, schizophrenia patients outscoring controls and patients with bipolar disorder in both visual and auditory modalities.

Kruskal-Wallis ANOVAs indicated a significant difference among patients with schizophrenia, bipolar disorder, and controls in the SIAPA visual modality [H (2) = 25.75, *p* < 0.001] and in the SIAPA auditory modality [H (2) = 22.25, *p* < 0.001]. As shown in Figure 3, patients with schizophrenia scored higher relative to the bipolar disorder and the control group in both visual and auditory modalities (*p*'s < 0.01).

**Figure 4:**
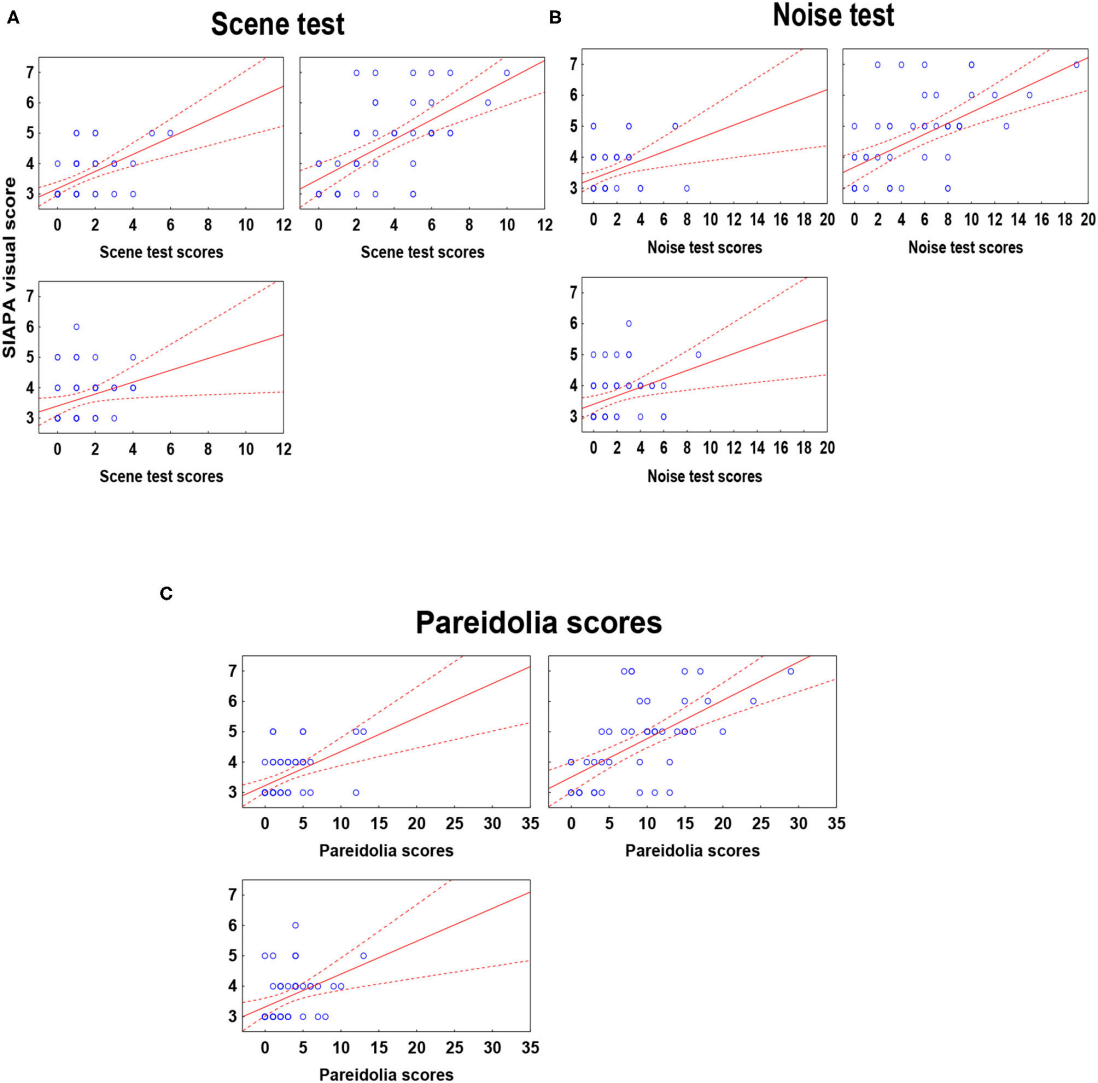
Correlations between SIAPA (Structured Interview for Assessing Perceptual Anomalies) visual scores and **(A)** scence, **(B)** test, and **(C)** pareidolia test results. The correlation coefficients are shown in Supplementary Table 2.

Supplementary Table 2 depicts correlations between the pareidolia scores and clinical measures (including SIAPA, PANSS, and YMRS). In schizophrenia, there were significant positive relationships between the scene, noise, and total pareidolia scores and the SIAPA visual scores, which survived Bonferroni correction (rs > 0.6, *p*'s < 0.001) (Figure 4). There were no significant correlations when the auditory SIAPA scores were included in the analysis, and the remaining correlations including PANSS and YMRS values did not reach the level of statistical significance (Supplementary Table 2).

## Discussion

In the current study, we have measured pareidolia in patients with schizophrenia, patients with bipolar disorder, and healthy controls. To our knowledge, this is the first study to concurrently compare pareidolia measures in these populations. Our results show that schizophrenia patients show more illusory perception than patients with bipolar disorder and healthy controls. Similarly, our results also show that patients with schizophrenia scored higher than patients with bipolar disorder and healthy controls in both visual and auditory modalities of the Structured Interview for Assessing Perceptual Anomalies (SIAPA). Furthermore, we found a positive correlation between scene, noise, and pareidolia tests and visual scores of the SIAPA measures, but not with PANSS and YMRS. In addition, our study has also some novel findings that are not reported in prior studies. For example, we found that pareidolia measures successfully differentiated schizophrenia from health controls and also differentiated schizophrenia from bipolar disorder. However, pareidolia measures were not strong predictors of classifying healthy controls from bipolar disorder. In addition, patients with BPD and SCZ scored similarly and mildly on depression scales because they were in clinically stable conditions [for discussion on these points, see (62)]. The depression scores were low in both groups (mild-subthreshold level according to the NIHCE 2019 criteria) indicating no clinically significant major depressive episode in bipolar and schizophrenia patient. It is a major strength of the study because co-morbid depression is a serious confounding factor that may interfere with test results.

Our findings are in agreement with prior studies. A recent study has also reported face pareidolia in patients with schizophrenia using the Giuseppe Arcimboldo food-plate stimuli, which are stimuli made out of food but the whole stimulus is usually perceived as a face (48). However, our results are different from those of Mavrogiorgou et al. (46). Mavrogiorgou et al. (46) found that schizophrenia patients show less pareidolia symptoms than healthy individuals. The discrepancy in findings between these studies and ours can be related to the differences in demographical variables of healthy controls and schizophrenia patients in these studies. Specifically, our controls have average age of 38, while healthy controls in the Mavrogiorgou et al. (46) study have an average age of 46. The difference in age in schizophrenia patients in our study and that of Mavrogiorgou et al. (46) is less pronounced. Our schizophrenia patents have average age of 40, while healthy controls in the Mavrogiorgou et al. (46) study have an average age of 43. These differences in age, especially in age, may perhaps explain the discrepancy in findings. Furthermore, the discrepancy in findings between our study and that of Mavrogiorgou et al. (46) can be related to differences in stimuli, as the Mavrogiorgou et al. (46) used self report while we have used several novel cognitive pareidolia measures consisting of blurred images of natural scenes intended to produce illusory face responses.

There is also some evolutionary explanation of pareidolia. The ability to make sense of a stimulus based on noisy or ambiguous sensory data is suggested to be an adaptive function of the brain. The tendency to infer agency from sensory noise is thought to have evolved to serve an important function in predatory threat detection (63), but in daily life, can yield perceptual errors, such as mistaking an object as a face. However, another interpretation is that pareidolia is related to our increased cognitive fluidity and prosocial behavior (64). To test the plausibility of both views, future work should use and correlate surveys that measure predatory threat detection and prosocial behavior, along with measures of pareidolia.

There are debates in the field on whether schizophrenia and bipolar disorder fall on the same continuum or are vastly different disorders (65, 66). Existing data on this topic is conflicting. As mentioned in Introduction, some studies found that schizophrenia patients are more impaired than bipolar disorder patients on several cognitive measures (67, 68), which is in agreement with our results. However, other studies argue that such disorders fall on a continuum, and are not thus markedly different (69). In line with this view, some studies found that patients with schizophrenia and bipolar disorder patients with psychosis symptoms are similarly impaired on several cognitive measures, in comparison to healthy controls (70–74).

As mentioned above, illusion and pareidolia can results from top-down processing deficits (15–19). The question is, how can we explain our findings that schizophrenia patients show more pareidolia than healthy controls and patients with bipolar disorder? Here, we argue that this is possibly due to more damage in the prefrontal cortex in schizophrenia than in bipolar disorder patients, as discussed above (10, 75). In other words, it is possible that the more severe prefrontal damage in schizophrenia patients lead to an impaired top-down processing, and this in turn, lead to illusion and pareidolia. However, this mechanism should be investigated in future studies.

### Limitations and Future Studies

Our study suffers from some limitations. For example, unlike prior studies, we did not include patients with bipolar disorder I and II (76, 77). Future work on pareidolia should include bipolar disorder patients with mania and hypomania. It is predicted that patients with bipolar disorder II may show reduced pareidolia than patients with bipolar disorder I. However, subgroups of schizophrenia with varying degrees of negative symptoms [i.e., deficit vs. non-deficit schizophrenia, (78, 79)] may not show any differences in measures of pareidolia. Further, future studies should also investigate pareidolia in individuals with schizotypal personality disorder as well as other patient groups with schizophrenia-related disorders, such as schizoaffective disorder. It is predicted that like schizophrenia, these patient groups may also show pareidolia. Other limitation of the study is we do not have enough information from participants regarding their racial/ethnic identification, and culture/geographic background, a measure of income, and socioeconomic status. In addition, another limitation is a larger number of bipolar disorders are on mood stabilizers than patients with schizophrenia. However, this is the case in almost all studies on schizophrenia and bipolar disorder, and it is often possible to match patient groups on their medication use.

Future studies should measure neural activations underlying the occurrence of pareidolia in patients with schizophrenia and bipolar disorder. Based on prior findings (80, 81), we predict that schizophrenia patients may show more fusiform area activation than patients with bipolar disorder during the performance of the scene test used in the task. Future work should investigate whether pareidolia is more common in psychotic bipolar disorder patients than in non-psychotic bipolar disorder patients. In addition, one study found that negative mood increases pareidolia in patients with Lewy body dementia (82). Future work should also investigate whether negative mood increase pareidolia in patients with schizophrenia and bipolar disorder. Future research should also investigate the impact of antipsychotics as well as other medications for the treatments of schizophrenia and bipolar disorder, such as lithium, on the occurrence of pareidolia.

## Data Availability Statement

The raw data supporting the conclusions of this article will be made available by the authors, without undue reservation.

## Ethics Statement

The studies involving human participants were reviewed and approved by Hungarian Scientific and Research Committee of the Medical Research Council ethics board (Budapest, Hungary). The patients/participants provided their written informed consent to participate in this study.

## Author Contributions

EA and DB developed the study concept. SK contributed to the study design. Testing and data collection were performed by SK and KC. SK and KC performed the data analysis and interpretation under the supervision of AM. AM drafted the paper. EA and AM provided critical revisions. All the authors acknowledged that they contributed equally in the current paper and approved the final version of the paper for submission.

## Conflict of Interest

The authors declare that the research was conducted in the absence of any commercial or financial relationships that could be construed as a potential conflict of interest.

## Publisher's Note

All claims expressed in this article are solely those of the authors and do not necessarily represent those of their affiliated organizations, or those of the publisher, the editors and the reviewers. Any product that may be evaluated in this article, or claim that may be made by its manufacturer, is not guaranteed or endorsed by the publisher.
