# Supplementary material for: Pareidolia in Schizophrenia and Bipolar Disorder
Source: Front Psychiatry. 2021 Dec 10;12:746734. doi: 10.3389/fpsyt.2021.746734 (PMC8702957; doi:10.3389/fpsyt.2021.746734)
Supplement: Supplementary file 1 [file Data_Sheet_1.PDF]

Pareidolia in Schizophrenia and Bipolar Disorder

Supplementary material

Tables

	Wilk's lambda	F	df	p	Squared Mahalanobis distances	Correctly classified cases
SCZ - control						
Scene	0.75	32.37	1, 98	< 0.001	1.29	72%
Noise	0.74	33.94	1, 98	< 0.001	1.36	73%
Pareidolia	0.72	37.28	1, 98	< 0.001	1.49	76%
SCZ - BPD						
Scene	0.76	31.55	1, 98	< 0.001	1.26	72%
Noise	0.79	25.68	1, 98	< 0.001	1.03	68%
Pareidolia	0.76	31.80	1, 98	< 0.001	1.27	73%
BPD - control						
Scene	0.99	0.14	1, 98	0.71	0.01	54%
Noise	0.98	1.26	1, 98	0.26	0.05	56%
Pareidolia	0.99	0.86	1, 98	0.36	0.03	57%

Table S1. *Discriminant function analysis.*

	SIAPA visual			SIAPA auditory			PANNS - P			PANNS - N			PANNS - G			YMRS		
	CO	SZ	BP	CO	SZ	BP	CO	SZ	BP	CO	SZ	BP	CO	SZ	BP	CO	SZ	BP
Scene	0.60*	0.66*	0.39	0.14	-0.07	0.10	-	0.24	0.07	-	0.10	0.14	-	0.00	0.21	-	0.01	0.07

Noise	0.38	0.62*	0.45	0.00	-0.04	0.09	-	0.23	-0.19	-	-0.03	-0.18	-	0.07	-0.09	-	0.01	0.04
Pareidolia	0.53*	0.65*	0.51*	0.10	-0.05	0.13	-	0.24	-0.07	-	0.01	-0.04	-	0.05	0.07	-	0.00	0.06

Table S2. *Correlations between the pareidolia scores and the clinical measures. * p < 0.001 (Bonferroni-corrected threshold of significance); CO – controls; SZ – schizophrenia; BP – bipolar disorder; SIAPA - Structured Interview for Assessing Perceptual Anomalies; PANSS - Positive and Negative Syndrome Scale; P – positive symptoms; N – negative symptoms; G – general symptoms; YMRS - Young Mania Rating Scale.*

Data

group	scene	face	noise	parei	SIAPA_vis	SIAPA_aud	PANSS_P	N	G	IQ
0	1	8	0	1	3	0				107
0	2	8	1	3	4	0				115
0	0	8	0	0	3	0				103
0	2	8	0	2	4	1				89
0	0	8	0	0	3	0				117
0	0	7	0	0	3	0				95
0	0	8	0	0	3	1				109
0	0	6	0	0	3	1				115
0	0	8	1	1	3	1				91
0	0	8	1	1	3	0				120
0	2	8	3	5	5	0				99
0	1	8	3	4	4	1				98
0	2	8	4	6	3	0				103
0	2	6	3	5	4	1				95
0	0	7	1	1	3	0				117
0	0	7	1	1	3	2				125
0	0	7	0	0	3	0				103
0	0	8	0	0	3	0				94
0	0	8	0	0	4	0				90
0	0	8	0	0	3	0				104
0	1	8	0	1	5	1				106
0	3	8	2	5	4	0				109
0	5	8	7	12	5	0				104
0	1	8	0	1	3	1				94
0	0	8	0	0	3	2				96
0	1	8	1	2	4	2				92
0	1	7	0	1	5	1				90

0	2	8	3	5	5	0				102
0	1	8	2	3	4	0				103
0	4	8	8	12	3	1				112
0	3	8	2	5	4	2				121
0	1	8	1	2	3	2				106
0	2	8	0	2	3	0				109
0	1	6	0	1	4	0				107
0	1	8	0	1	3	0				94
0	0	8	1	1	3	0				90
0	0	8	0	0	3	0				98
0	0	8	0	0	3	0				102
0	1	6	0	1	4	1				114
0	1	8	1	2	3	0				103
0	1	8	2	3	3	1				94
0	4	8	2	6	4	2				95
0	6	8	7	13	5	0				91
0	0	8	1	1	3	0				102
0	0	8	0	0	3	0				114
0	3	8	0	3	3	1				105
0	0	7	0	0	3	2				101
0	0	7	0	0	3	0				89
0	1	8	4	5	3	0				94
0	1	7	3	4	4	0				93
1	4	8	8	12	5	2	8	8	21	95
1	3	8	7	10	6	1	12	12	45	97
1	6	8	9	15	5	0	14	14	39	104
1	6	8	9	16	5	1	23	19	39	105
1	5	8	10	15	7	0	10	15	24	100
1	4	8	7	11	5	2	8	19	45	90
1	3	5	6	9	4	1	11	21	48	94
1	4	7	6	10	5	0	15	23	37	125
1	3	8	6	9	6	1	14	18	41	101
1	3	8	8	11	5	4	14	14	37	85
1	2	7	5	7	5	4	18	16	23	93
1	5	7	10	15	6	2	23	18	45	102
1	6	8	12	18	6	3	22	14	50	126
1	7	8	10	17	7	0	20	16	53	96
1	6	8	9	15	5	2	18	16	36	86
1	5	8	8	13	4	2	16	21	41	91
1	6	8	9	15	5	2	12	22	33	102
1	7	8	13	20	5	2	23	25	52	94
1	9	8	15	24	6	4	23	27	54	104
1	2	6	1	3	4	2	21	25	48	110
1	2	6	1	3	3	4	12	18	43	98
1	2	6	3	5	5	3	14	20	44	96
1	1	8	0	1	3	3	17	21	39	86
1	2	8	6	8	7	2	19	23	38	97
1	0	7	0	0	3	3	12	13	42	80
1	0	8	3	3	3	4	16	17	46	85
1	0	8	0	0	3	0	20	21	47	103
1	4	8	0	4	5	1	22	24	49	98
1	5	8	6	11	5	0	21	19	56	92
1	3	8	8	11	3	0	27	25	63	91
1	2	8	8	10	5	0	9	8	20	100

1	2	8	3	5	4	4	10	8	30	93
1	2	7	2	4	4	0	10	8	26	96
1	1	7	0	1	3	1	11	12	29	104
1	1	7	0	1	3	1	9	14	27	108
1	0	8	0	0	4	0	8	19	25	104
1	0	7	0	0	3	1	12	21	41	96
1	0	8	0	0	4	3	12	20	44	99
1	1	8	0	1	3	4	13	18	46	95
1	1	8	1	2	4	0	16	16	49	101
1	3	7	4	7	7	0	18	19	40	110
1	1	6	3	4	3	0	17	21	43	121
1	0	5	3	3	3	2	25	20	62	95
1	6	8	2	8	5	0	17	15	32	90
1	7	8	3	10	5	4	13	19	34	95
1	6	8	2	8	7	0	14	23	30	96
1	5	8	4	9	3	4	21	20	44	89
1	5	8	8	13	3	3	25	26	46	87
1	6	8	8	14	5	2	12	21	35	106
1	10	8	19	29	7	0	10	12	31	109
2	2	8	2	4	4	1	13	11	30	104
2	1	8	0	1	4	0	11	14	26	110
2	0	8	0	0	3	1	14	18	28	102
2	0	8	0	0	3	1	18	16	31	100
2	0	8	0	0	3	0	21	17	34	98
2	3	8	0	3	3	0	24	19	36	110
2	2	7	1	3	4	0	21	22	38	104
2	4	7	6	10	4	1	20	21	41	102
2	2	7	2	4	5	1	10	13	49	106
2	2	8	2	4	4	0	12	15	36	83
2	3	7	4	7	4	2	13	17	44	97
2	2	8	6	8	3	3	8	10	30	99
2	1	7	6	7	3	1	9	9	27	95
2	1	7	3	4	4	0	7	7	29	101
2	0	6	2	2	4	0	7	7	23	97
2	0	7	0	0	5	0	12	14	35	99
2	0	8	0	0	3	1	15	17	38	103
2	2	6	0	2	3	0	11	13	25	105
2	1	7	3	4	6	0	10	12	24	110
2	0	8	0	0	3	1	15	15	45	104
2	1	8	0	1	3	0	19	19	49	102
2	2	8	0	2	4	2	21	21	57	96
2	4	8	0	4	4	1	24	21	68	90
2	3	8	1	4	4	0	25	16	73	91
2	1	8	1	2	3	0	21	14	54	94
2	1	8	4	5	3	1	20	9	43	87
2	0	8	0	0	3	1	23	9	49	113
2	0	7	2	2	3	2	22	11	40	125
2	2	8	1	3	3	0	12	10	35	130
2	1	7	3	4	5	0	14	13	39	102
2	4	8	5	9	4	0	15	14	33	105
2	2	6	3	5	4	1	11	8	26	87
2	1	7	1	2	3	0	9	7	24	95
2	0	8	0	0	3	0	13	12	37	93
2	0	8	0	0	3	0	18	17	39	90

2	0	8	0	0	3	0	12	10	26	98
2	0	8	0	0	3	0	14	15	23	103
2	1	8	0	1	3	0	21	19	47	109
2	1	8	0	1	3	2	24	17	49	94
2	2	8	0	2	4	0	25	23	52	90
2	1	8	0	1	3	2	26	25	57	108
2	1	8	5	6	4	0	23	21	46	112
2	4	8	9	13	5	0	25	24	50	95
2	2	8	4	6	4	0	19	17	37	92
2	1	8	3	4	5	2	21	19	41	90
2	0	7	4	4	4	0	23	22	46	103
2	0	7	1	1	3	0	18	17	30	106
2	0	7	1	1	5	0	19	18	36	103
2	0	8	1	1	3	0	27	26	63	96
2	1	7	2	3	3	2	8	7	20	97
